# Role of magnetic resonance spectroscopy in cerebral glutathione quantification for youth mental health: A systematic review

**DOI:** 10.1111/eip.12833

**Published:** 2019-05-31

**Authors:** Emily Fisher, John Gillam, Rachel Upthegrove, Sarah Aldred, Stephen J. Wood

**Affiliations:** ^1^ School of Sport, Exercise and Rehabilitation Sciences University of Birmingham Edgbaston UK; ^2^ Orygen the National Centre of Excellence in Youth Mental Health Melbourne Victoria Australia; ^3^ Centre for Youth Mental Health University of Melbourne Melbourne Victoria Australia; ^4^ Institute for Mental Health University of Birmingham Edgbaston UK; ^5^ Department of Psychiatry University of Birmingham Birmingham UK

**Keywords:** glutathione, magnetic resonance spectroscopy, youth mental health

## Abstract

**Aim:**

Oxidative stress is strongly implicated in many psychiatric disorders, which has resulted in the development of new interventions to attempt to perturb this pathology. A great deal of attention has been paid to glutathione, which is the brain's dominant antioxidant and plays a fundamental role in removing free radicals and other reactive oxygen species. Measurement of glutathione concentration in the brain in vivo can provide information on redox status and potential for oxidative stress to develop. Glutathione might also represent a marker to assess treatment response.

**Methods:**

This paper systematically reviews studies that assess glutathione concentration (measured using magnetic resonance spectroscopy) in various mental health conditions.

**Results:**

There is limited evidence showing altered brain glutathione concentration in mental disorders; the best evidence suggests glutathione is decreased in depression, but is not altered in bipolar disorder. The review then outlines the various methodological options for acquiring glutathione data using spectroscopy.

**Conclusions:**

Analysis of the minimum effect size measurable in existing studies indicates that increased number of participants is required to measure subtle but possibly important differences and move the field forward.

## 1 INTRODUCTION

There is now extensive evidence that oxidative stress, defined as a disturbance in the balance between the production of reactive oxygen species and antioxidant defences (Betteridge, 2000) plays a role in the pathophysiology of many psychiatric disorders, including depression, bipolar disorder, anxiety and schizophrenia (Smaga et al., 2015). For example, depressed patients display increased markers of oxidative stress in plasma with an associated decrease in total antioxidant capacity (Gałecki, Szemraj, Bieńkiewicz, Florkowski, & Gałecka, 2009) (Yumru et al., 2009), and similar findings have been reported in anxiety (Atmaca, Kuloglu, Tezcan, & Ustundag, 2008) and schizophrenia (Akiibinu, Ogundahunsi, & Ogunyemi, 2012; Dietrich‐Muszalska, Olas, Głowacki, & Bald, 2009). The result of this oxidative stress is cellular damage, with consequent impacts on cell function or even cell death. The presence of markers of oxidative stress across diagnostic groups suggests some common pathophysiological process that might be amenable to universal treatment, an approach that would be particularly helpful in the early stages of mental illness when specific diagnoses have not yet crystalized (McGorry, Hickie, Yung, Pantelis, & Jackson, 2006).

The primary focus of much research in this area has been glutathione (GSH). GSH is the brain's dominant antioxidant, and plays a fundamental role in removing free radicals and other reactive oxygen species (Wood, Yücel, Pantelis, & Berk, 2009). Both reduced (GSH) and oxidized (GSSG) GSH are present in cerebral tissue. Reduced GSH is converted to GSSG either directly or through catalysis by glutathione peroxidase (GPx) (Xin et al., 2016), upon interaction with a radical species. This process is protective for cells and intracellular cellular components against damage. GSSG is subsequently reduced back to GSH, a reaction catalysed by glutathione reductase (GR), and in this way GSH contributes to regulation and maintenance of cellular redox status (Lushchak, 2012). The ratio of the redox active couple (GSH‐GSSG) can be used directly to measure oxidative stress, but precise quantification of this ratio is challenging, even in blood measures, so that GPx and GR assessment are frequently used instead (Xin et al., 2016). In post‐mortem studies of the prefrontal cortex (PFC) of patients and non‐psychiatric controls, GSH, GPx and GR alterations were associated with bipolar disorder, major depressive disorder and schizophrenia (Gawryluk, Wang, Andreazza, Shao, Yatham et al., 2011; Gawryluk, Wang, Andreazza, Shao, & Young, 2011). While direct measurement of these metabolites can be conducted in brain post‐mortem, in vivo quantification generally remains extremely challenging; however, GSH concentration in the brain can be assessed using magnetic resonance spectroscopy (MRS).

This review therefore aims to integrate the results of published studies employing MRS measurement of GSH in the brains of those presenting with mental illness, particularly young people. Two main questions will be addressed in this review:

1. Is perturbed GSH reliably associated with indicators of mental illness in young people, such as diagnosis, symptoms or functioning?
2. What are the strengths and weaknesses of the various approaches to the in vivo quantification of GSH concentration using MRS?

These two questions are of importance to ongoing and planned investigations regarding aetiology and prognosis in youth mental health. Simultaneously, the optimal GSH measurement protocols that utilize MRS are paramount. In order to address these questions, a systematic review was conducted selecting those publications that contained human in vivo neurological MRS studies in which cerebral GSH concentration was a target metabolite considered as a possible marker of mental health conditions.

## 2 METHODS

**Figure 1:**
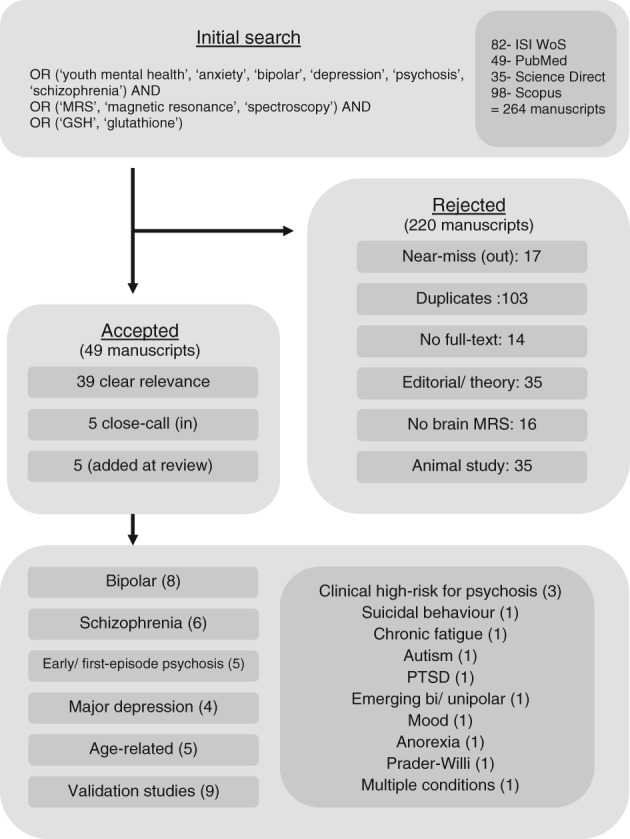
Flow diagram representing the database search with relevant accepted manuscripts considered in this investigation. PTSD, post traumatic stress disorder

This systematic review followed PRISMA guidelines (Moher et al., 2009) (Figure 1). However, due to the recent and emerging nature of the outcome measures, no quantitative meta‐analysis was conducted on the resulting data.

### 2.1 Databases and search terms

The review aims to provide information that will inform the design and parameters of an investigation into the role of GSH in the quantification of neurological markers associated with conditions relating to youth mental health. Such a study would require that optimal parameters to assess GSH concentration in small regions of interest within the brain, using standard, routine, clinical infrastructure currently available. Studies considered within the present review were limited to those published after 2000 in order to restrict results to technically relevant procedures.

Four databases were identified for search, three of which provided general coverage (Web of Science, ScienceDirect and Scopus) and one of which was medically focussed (PubMed). Records were requested from each database (as of July 16, 2018) using the same search terminology and using a breadth‐first approach to ensure sufficient coverage. Three sub‐strings were required, each arising from a range of alternatives: the population of interest (“youth mental health” OR “anxiety” OR “bipolar” OR “depression” OR “psychosis” OR “schizophrenia”), the measurement technique (“MRS” OR “magnetic resonance” OR “spectroscopy”) and the comparative measure, in this case a target metabolite (“GSH” OR “Glutathione”), were required (AND) in the title or abstract (where possible) of the publications returned.

The database search returned 264 studies in total (WoS:82, Sd:35, Sc:98, PM:49) 103 of which were identified as duplicate studies (ie, the same paper was present within the studies returned from two or more data bases). The remaining studies were assessed for relevance. Inclusion criteria were reasonably broad, requiring human study‐participants undergoing in vivo MRS of brain GSH with quantitative outcomes and accessible full‐text material. From the considered studies, 108 were rejected as they did not meet these criteria, while nine were not assessed (as they were not appropriate for the analysis) leaving 49 studies of relevance to this review.

## 3 RESULTS

Relevant features (deemed important variables for the review) extracted from studies are shown in Table 1.

**Table 1:** In vivo MRS of GSH for mental health: summary of findings

Condition	Brain region	MR‐Sq	Study and main finding	#Part.	*d*_min_	Other GSH‐specific correlations
BD	ACC	3 T PRESS	(Lagopoulos et al., 2013) No difference in GSH between patients and controls: (*t*(115) = 1.253, *P* = .213).	53 (BD) 51 (HC)	0.6	GSH vs YMRS: (*ρ* = −0.198; *P* = .214; N = 41) GSH vs HDRS: (*ρ* = 0.127; *P* = .385; N = 49) GSH vs age of onset: (*ρ* = −0.09; *P* = .522; N = 53)
						GSH vs duration (*ρ* = −0.125; *P* = .374; N = 53).
		3 T PRESS	(Chitty, Lagopoulos, et al., 2013) Risky drinking in patients showed less GSH than non‐risky: (*t*(48) = 2.44, *P* = .015).	33 (BD) 17 (HC)	0.9	High alcohol use disorders identification test score negatively correlated with GSH in BD subjects (*r* = −0.478, *P* = .005).
		3 T PRESS	(Chitty, Lagopoulos, Hickie, & Hermens, 2015a, 2015b) Change in alcohol use, smoking and age predict changes in GSH (at 15 months): (*F*(3, 26) = 3.69, *P* < .05). Not controlled as comorbid.	30 (BD)	0.5	GSH vs alcohol frequency: *r* = −0.381, *P* < .05 GSH vs smoking frequency: *r* = −0.367, *P* < .05
		3 T J‐PRESS	(Soeiro‐de‐Souza et al., 2016) No difference in GSH between euthymic BD and controls. Lac ∝ GSH in BD patients	50 (BD) 38 (HC)	0.6	Lac vs GSH Patients: (*B* = 0.20, *t* = 3.2, *P* = .003 [0.07, 0.33]) Controls:(*B* = 0.17, *t* = 0.64, *P* = .11 [0.04, 0.39])
	ACC + Hip	3 T PRESS	(Chitty, Lagopoulos, Hickie, & Hermens, 2014) No difference in GSH in either region between BD and Controls. Differences mediated by drinking and smoking.	64 (BD) 49 (HC)	0.5	GSH_Hip_ vs risky drinking (BD): (*r* = 0.489, *P* < .021) GSH_ACC_ vs smoking (BD): (*t*(53) = 4.162, *P* < .001)
	l‐Hip	3 T PRESS	(Chitty et al., 2015b) Controls: mismatch negativity correlated with GSH decrease in both temporal sites. *r*_left_ = −0.542 [−0.8, −0.06] *r*_right_ = −0.374 [−0.67, 0.07] No association in BD patients.	28 (BD) 22 (HC)	0.8	GSH vs left‐MMN (*r* = 0.068, *P* = .74, 95% [−0.36, 0.69])
						GSH vs right‐MMN (*r* = −0.057, *P* = .78, 95% [−0.52, 0.73]).
	OCC + mPFC	3 T SPECIAL	(Godlewska, Yip, Near, Goodwin, & Cowen, 2014) No difference between BP and control in either region (for GSH or other metabolites).	13 (BD)11 (HC)	1.2	
	ACC + OCC	7 T STEAM	(Masaki et al., 2016) After treatment: No change in GSH_OCC_ Decrease in GSH_ACC_ (*P* = .033) GSH_ACC plc._ =1.31 土 0.043 GSH_ACC Ebselen_ = 1.17土0.07	20 (HC)	0.6	
Schiz. (SZ)	ACC	4 T STEAM	(Terpstra et al., 2005) STEAM was within uncertainty of edited spectra in in vivo tests (*P* = .4). GSH levels of patients not different from controls (*P* = .4, differences >10%).	13 (SZ)9 (HC)	1.3	GSH_pat_ = 1.6土0.2 GSH_cont._ = 1.5土0.3
		MEGA‐ PRESS				
		7 T STEAM	(Brandt et al., 2016) GSH differences between patients and controls under the age of 40: [*t*(25) = −2.47, *P* = .021]	24 (SZ) 24 (HC)	0.8	GSH not correlated with age Overall no GSH difference between patients and controls.
	ACC + LI + VC	7 T STEAM	(Kumar et al., 2018) GSH lower in patients vs healthy controls‐ only in ACC voxel ACC *P* = .008 LI *P* = .784 VC *P* = .464	28 (SCH) 45 (HC)	0.7	GSH and glutamine correlated in all three voxels GSH vs ACC: *r* = 0.56 GSH vs LI: *r* = 0.80 GSH vs VC: *r* = 0.65
	mPFC	1.5 T PRESS	(Do, Trabesinger et al.) Cerebrospinal fluid GSH sample showed 27% decrease in patients (*P* < .05). MRS showed 52% decrease in patients (*P* < .0012).	14 (SZ) 14 (HC)	1.1	
	pMFC	3 T MEGA‐ PRESS	(Matsuzawa et al., 2008) No significant difference between patients and controls.	20 (SZ) 16 (HC)	1.0	For patients GSH correlated to negative symptoms SANS and BPRS (*r* = −0.68, *P* < .001) and related to trail making test A (*P* < .05).
	Imag.	4 T proton echo planar spectroscopic imaging	(Bustillo et al., 2011) No GSH‐specific hypothesis tested.	30 (SZ) 28 (HC)	0.8	
Major Depression (MD)	OCC, bilat.	3 T MEGA‐ PRESS	(Lapidus et al., 2014) GSH negatively correlated to anhedonia severity: (*r* = −0.55, *P* = .01).	11 (MD) 10 (HC)	1.3	MDD sample in isolation showed associations between anhedonia and GSH: (*r* = −0.53, *P* = .09). No associations between fatigue severity and GSH
	OCC	3 T SPECIAL	(Godlewska, Near, & Cowen, 2015) GSH was decreased in depressed patients *F* = 5.10, *P* = .028 *F* = 4.28, *P* = .042 (con. Age/sex)	39 (MD) 31 (HC)	0.7	
		3 T PRESS	(Freed et al., 2017) GSH decreased in MD patients' vs HCs *P* = .04	19 (MD) 8 (HC)	1.3	No correlation between GSH and anhedonia, MD severity, or onset
	Imag.	3 T MRSI	(Li et al., 2016) In left putamen, GSH decreased in patients (*P* = .044) Patient increase post therapy not significant.	16 (MD) 10 (HC)	1.2	GSH/tCr_pat._ = 0.23士0.06 GSH/tCr_cont._ = 0.28士0.05
Early Psych. (FEP/EP)	Temp	3 T PRESS	(Berger et al., 2008) Bilateral GSH increase in treatment group response (*F*_1,12_ = 6.1, *P* = .03) No longer significant when affective psychotic patients removed.	24 (FEP)	0.6	PANSS negative symptom change negatively correlated with GSH (*r* = −0.57, *P* = .041). Percent change in GSH and Glutamate/Glutamine correlated: (*r* = 0.64, *P* = .01)
		3 T PRESS	(Wood et al., 2009) GSH 22% higher in patients than controls: (*F*_1,46_ = 4.7, *P* = .035). No difference in other tests: hemisphere (*P* = .137), group‐by‐hemisphere (*P* = .513).	30(FEP) 18(HC)	0.9	Patients not responding to topical niacin show 35% higher GSH than responders (*F*_1,28_ = 5.1, *P* = .007).
	mPFC	3 T SPECIAL	(Monin et al., 2015) Potential dependence between GSH levels and white matter integrity during PFC developments.	30 (EP) 40 (HC)	0.7	Controls: GSH correlated to general FA (*r* = 0.34, *P* = .03) and functional connectivity (*r* = 0.40, *P* = .01). Patients controlled for medication and duration: GSH correlated to general FA (0.31, *P* = .01).
		3 T SPECIAL	(Xin et al., 2016) GSH decrease (*P* = .006) in glutamate‐cysteine ligase catalytic high‐risk (1.15 土 0.17) compared to low‐risk (1.34土0.8).	25 (EP) 33 (HC)	0.8	GSH_mPFC_ correlated to GSH_blood_ in controls (*P* = .021) but not in patients (*P* = .39).
CHR for psychosis	mPFC	3 T PRESS	(Hafizi et al., 2018) GSH and TSPO radioligand significant negative association in healthy volunteers, but not clinical high‐risk group—indicative of an abnormal interaction TSPO expression and redox status in CHR group	27 (CHR) 21 (HC)	0.9	mPFC GSH and [^18^F]FEPPA VT (radioligand of TSPO) not sig. Different between groups.
		3 T PRESS	(Da Silva, Hafizi et al. 2018a) No sig difference between mPFC GSH in drug‐naïve patients vs healthy controls	30 (CHR) 27 (HC)	0.8	No sig correlations between cerebral GSH and clinical and neuropsychological measures No sig difference between GPx activity and CHR vs HC (*F* = 0.15, *P* = .70)
						Significant effect lifetime cannabis use in GPx activity (*F* = 7.41, *P* = .01)
		3 T PRESS	(Da Silva et al., 2018) No sig difference between mPFC GSH in drug‐naïve patients vs healthy controls	27 (CHR) 16 (HC)	0.9	No differences between microglial activation and GSH between groups
	ACC + Striat.	3 T PRESS	(Demro et al., 2017) GSH_ACC_ was shown to correlate significantly with P3 (Grandiosity, negative) and P5 (Disorganized Communication, positive) as measured by the SIPS GSH_STR_ was shown to (negatively) correlate significantly only to Grandiosity (P3)	12 (CHR)	0.7	GSH correlation with SIPS: P1: *r*_ACC_ = −0.578 (0.062), *r*_STR_ = −0.566 (0.088) P2: *r*_ACC_ = −0.074 (0.828), *r*_STR_ = −0.474 (0.167) P3 *r*_ACC_ = −0.673 (0.023), *r*_STR_ = −0.775 (0.009) P4: *r*_ACC_ = −0.259 (0.441), *r*_STR_ = −0.409 (0.241) P5: *r*_ACC_ = 0.645 (0.032), *r*_STR_ = 0.138 (0.704) Positive symptom sum: *r*_ACC_ = −0.134 (0.695), *r*_ACC_ = −0.550 (0.099)
Age‐related (AD, Deprs‐ at.risk, sleep‐ apnea, and MCI)	Th	3 T PRESS	(Duffy et al., 2015) Older patients at risk of DEP taking placebo had larger GSH/Cr (*t* = 2.0, *P* = .049).	51 (DEP) (28 treat+ 23 plac.)	0.8	Increased GSH_Th_ associated with worsening symptoms (*r* = 0.43, *P* = .043)
	ACC	3 T PRESS	(Duffy et al., 2015) Older patients at risk of DEP: increased GSH in the ACC (*t* = 2.7, *P* = .012)	58 (DEP) 12 (HC)	0.9	Depressed patients showed a correlation between HADS symptoms and GSH/Cr (*r* = 0.28, *P* = .035). Depressed patients showed a negative correlation between verbal learning and GSH/Cr (*r* = −0.28, *P* = .04)
		3 T PRESS	(Duffy et al., 2016) GSH/Cr correlated with decreased executive function.	24 (ARD)	0.6	GSH_ACC_ vs Oxygen desat: *r* = −0.54, *P* = .007 GSH_ACC_ vs apnea‐hypopnea: *r* = .42, *P* = .050 GSH_ACC_ vs response inhib: *r* = −.49, *P* = .015. GSH_ACC_ vs set shifting: *r* = −0.43, *P* = .37.
	ACC + PCC	3 T PRESS	(Duffy et al., 2014) MCI patients showed increased levels of GSH in the cingulate: GSH_ACC_ (*t* = −2.2, *P* = .03) GSH_PCC_ (*t* = −2.9, *P* = .05)	54 (MCI) 41 (HC)	0.6	MCI GSH_ACC_: 0.47 土 0.15 MCI GSH_PCC_: 0.37土 0.07
						Control GSH_ACC_: 0.41土 0.10
						Control GSH_PCC_: 0.29土 0.05
	Vari.	3 T MEGA‐ PRESS	(Mandal, Tripathi, & Sugunan, 2012) Female AD showed decreased GSH in RFC (*P* = .003) compared to young controls. Male AD showed decreased GSH in the LFC	25 (ym) 20 (yf) 9 (om) 6 (of)	~1.3	GSH_LFC_ different from GSH_RFC_ in young female (*P* = .02) and male (*P* = .001) subjects. GSH_LFC_ vs GSH_RFC_: young females (*r* = 0.641, *P* = .004)
			(*P* = .05) compared to young controls. Gender differences in GSH distribution evident.	5 (mMCI) 6 (fMCI) 7 (mAD) 7 (fAD)		GSH_LPC_ vs GSH_RpC_: young females (*r* = 0.797, *P* = .000) GSH_LFC_ vs GSH_LPC_: young males (*r* = 0.481, *P* = .032) (Healthy young males/females (ym/yf); healthy older males/females (om/of); males/females with mild cognitive impairment and Alzheimer's disease.)
Mult.	ACC	3 T PRESS	(Hermens, Lagopoulos, Naismith, Tobias‐Webb, & Hickie, 2012) Clustering of patients based on metabolites: 3 subgroups. GSH responsible for cluster 2.	37 (DD) 29 (BP) 22 (PD) 25 (HC)	N/A	Discriminant function 2 (40% variance) characterized by GSH/Cr (*r* = −0.753).
Suicidal behaviour	dPFC	3 T SPECIAL	(Jollant, Near, Turecki, & Richard‐Devantoy, 2016) No association was found between GSH and suicidal behaviour (*F* = 0.5, *P* = .6), but associations with other metabolites identified.	15 (SA) 10 (PC) 33 (HC)	~1.0	GSH_dPFC_ Suicide Attempters: 0.24土0.03 GSH_dPFC_ Patient Controls: 0.23 土 0.02 GSH_dPFC_ Healthy Controls: 0.23土0.03
Chronic fatigue	OCC + Imag.	3 T MEGA‐ PRESS/ MRSI	(Shungu et al., 2012) GSH deficits in patients with CFS and MDD relative to controls: (*F*_2,40_ = 15.93; *P* < .001)	15 (CFS) 15 (MD) 13 (HC)	1.0	GSH was inversely correlated with ventricular lactate (*r* = −0.545, *P* = .001) and a range of key indices of physical health.
Autism	Basal ganglia + dPFC	3 T PRESS	(Durieux et al., 2016) No GSH difference observed between patients and controls.	21 (ASD) 29 (HC)	0.8	Correlation between GSH and Autism spectrum disorder was observed in either region.
PTSD	ACC + dLPFC	3 T MEGA‐ PRESS	(Michels et al., 2014) Observed GSH levels 22.73% higher in patients than controls (*F* = 5.757, *P* = .025)	12 (PTS) 17 (HC)	1.1	GSH_ACC_: PTSD = 0.15 ± 0.03, Non = 0.11 ± 0.03 (*d* = 1.33) GSH_dLPFC_: = 0.14 ± 0.03, Non = 0.11 ± 0.03 (*d* = 1.00)
Emerging Unipolar/Bi polar	ACC	3 T PRESS	(Naismith et al., 2014) GSH not associated with unipolar/bipolar differentiation (*t* = 1.15, *P* = .255).	53 (EBD)	0.4	GSH not associated with sleep midpoint (*r* = 0.211, *P* = .151)
Mood	ACC + HIPP	3 T PRESS	(Hermens et al., 2018) Positive correlations between Fractional Anisotropy in the stria terminalis and GSH_ACC_ were found across groups (*r* = 0.215, *P* < .01). DEP or BD in combination with decreased GSH_ACC_ was associated with reduced FA.	94 (DEP) 76 (BD) 59 (HC)	0.2	Decreased white matter integrity was associated with decreased GSH_HIPP_.
Anorexia Nervosa	ACC + OC + PUT	7 T STEAM	(Godlewska et al., 2017) GSH levels unchanged between groups.	13 (AN) 12 (HC)	1.2	AN (SEM) HC (SEM) p‐value GSH_ACC_ 1.19 (0.07) 1.27 (0.10) 0.38 CRLB 10.2 (2.9) 8.9 (2.8) GSH_OCC_ 0.95 (0.03) 0.94 (0.04) 0.85 CRLB 10.5 (3.0) 10.67 (3.08) GSH^PUT^ 1.51 (0.45) 1.10 (0.05) 0.43 CRLB 15.0 (4.5) 15.4 (4.6)
Prader‐Willi Synd.	ACC + *P*‐OCC	‐ MEGA‐ PRESS	(Rice, Lagopoulos, Brammer, & Einfeld, 2016) No between‐group differences were observed for GSH.	15(PWS) 15 (HC)	1.0	
.Validation studies	mPFC	7 T PRESS	(Choi et al., 2010)			Optimized PRESS with sub‐TE pair showed improved selectability of coupled metabolites (eg, Glu, Gln, GSH).
	mPFC + rPFC	7 T J‐PRESS	(An et al., 2015)			TE‐optimized J‐PRESS was shown to minimize NAA signals while retaining GSH peak resolution.
	Hipp	3 T semi‐LASER	(Bednařík et al., 2015)			Using a short‐echo sequence with 5 minutes averaging the GSH CRLB was kept below 30% in a 4 mL voxel at 3 T.
	ACC	7 T PRESS	(Lally et al., 2016)			Intra Class Correlation (ICC) using TE‐optimized PRESS both within sessions (ICC > 0.7) and between sessions (ICC > 0.6) showed good repeatability. GSH negatively associated with age (*r* = −0.37, *P* < .05).
	ACC + PCC	3 T STEAM	(Wijtenburg et al., 2014)			Short‐TE phase rotation STEAM at 3 T showed excellent reproducibility for GSH: absolute reliability: SEM < 9.9%, relative reliability: ICCs 0.42‐0.51
	Midline parietal	3 T HERMES	(Saleh et al., 2016)			HERMES scanning protocol showed excellent separation of GABA and GSH. Results agree with MEGA‐PRESS, achieving similar signal‐to‐noise ratio in half the time.
	mPFC	7 T PRESS	(Choi et al., 2010)			Optimized PRESS sequence showed lower CRLBs of Gln and GSH than with STEAM.
		3 T SPECIAL	(Schubert, Kühn, Gallinat, Mekle, & Ittermann, 2017)			Short‐TE SPECIAL sequence was used to measure MRS spectra at 3 T in 21 healthy adults. GSH was detected with low uncertainty (CRLB < 30%) in only 16 cases.
		3 T J‐PRESS	(Jensen, Auerbach, Pisoni, & Pizzagalli, 2017)			Test–retest reliability of metabolite quantification was assessed in a 3 T shortened J‐resolved MRS sequence in healthy adolecents. GSH demonstrated satisfactory reliability with a score of 8.8–4.1%.

Abbreviations: ACC, anterior cingulate cortex; AD, Alzheimer's disease; AN, anorexia nervosa; ARD, age‐related disorder; ASD, autism spectrum disorder;BD, bipolar disorder; BP, bipolar disorder; BPRS, brief psychiatric rating scale; CAT, catalase; CFS, chronic fatigue syndrome; CRLB, cramer‐rao lower bound; CHR, clinical high risk; CRLB, Cramer Rao lower bound; DD, depressive disorder; DEP, depression; dLPFC, dorsal left prefrontal cortex; dPFC, dorsolateral prefrontal cortex; EBD, emerging bipolar disorder; EP, early psychosis; EPI, echo‐planar imaging; FA, fractional anisotropy; fAD, females with Alzheimer's disease; FEPPA, tracer; fMCI, females with mild cognitive impairment; FSL, FMRIB software library; GSH, glutathione; GCL, glutamate cysteine ligase; GABA, gamma‐aminobutyric acid; GSSG, glutathione disulphide; HADS, hospital anxiety and depression scale; HC, healthy control; HDRS, hamilton depression rating scale; HERMES, Hadamard encoding and reconstruction of mega‐edited spectroscopy; HIPP, hippocampus; ICC, inferior colliculus; J‐PRESS, J‐resolved spectroscopy; LFC, left frontal cortex; LI, left insular; mAD, males with Alzheimer's disease; MCI, mild cognitive impairment; MD, major depression; MDA, malondialdehyde; MDD, major depressive disorder; mMCI, males with mild cognitive impairment; MMN, mismatch negativity; MRS, magnetic resonance spectroscopy; MRSI, magnetic resonance spectroscopy imaging; mPFC, medial prefrontal cortex; NAA, n‐acetyl aspartate; OCC, occipital cortex; OCD, obsessive compulsive disorder; PANSS, positive and negative symptom scale; PC, patient controls; PD, psychotic disorder; PFC, prefrontal cortex; pMFC, posterior medial frontal cortex; PTS, post traumatic stress; PTSD, post traumatic stress disorder; PRESS, point‐resolved spectroscopy; PUT, putamen; PWS, prader‐willi syndrome; RFC, right frontal cortex; SA, suicide attempters; SANS, scale for assessment of negative symptoms; SOD, superoxide dismutase; SEM, standard error of the mean; SIPS, structured interview for psychosis‐risk syndromes; STEAM, stimulated echo acquisition model; SZ, schizophrenia; TBARS, thiobarbituric acid; TE, echo time; THC, tetrahydrocannabinol; TM, mixing time; TSPO, translocator protein; VC, visual cortex; VT, total distribution volume; YMRS, young mania rating scale.

Summary of findings, describing the clinical group, brain region of interest, magnetic resonance sequence used, study reference and main findings, number of control vs clinical participants, dmin (the minimum effect size that could be founds significant given the cohorts in each study), and other GSH‐specific correlations that are not directly related to the outcomes of the paper, but important nonetheless.

Those studies that included quantitative comparisons (either tests for significant differences or regression analysis) were rated according to the minimum effect size required to achieve a power of 0.8, given a significance value of less than 0.05. Using a simplified simulation,^1^ multiple realizations of participants were drawn from either:

- Two unit‐variance normal distributions with mean separation *d*_*μ*_ = *μ*_1_ − *μ*_1_, or
- A two‐element, zero‐mean, multivariate normal with covariance $\sum =\left[\begin{array}{cc} 1 & d_{\sigma }\\ d_{\sigma } & 1 \end{array}\right]$.

The effect size (*d*_*μ*_) or covariance (*d*_*σ*_) was then increased from zero until at least 80% of 1 × 10^4^ trials returned a result with a two‐tailed significance of less than 0.05. Results are shown in Table 1 as *d*_min_ (where *d*_*σ*_ is only shown for single‐group studies).

### 3.1 Non‐quantitative outcomes

#### 3.1.1 GSH and cerebral GSH Concentration

Given that GSH is the major free radical scavenger within the brain, that the GSH redox couple have been associated with psychiatric disorders, and that GSH may be directly measured using sophisticated MRS sequences in vivo within patient brains, a number of studies have been performed that probe this connection. However, due to the fact that there is considerable heterogeneity in methods for MRS data acquisition, most studies differ in voxel size and placement, parameters and post‐processing, and also the quantitative measures used to assess study outcomes. Therefore, it is not feasible to perform quantitative analysis on acquired results. However, there are a number of conclusions that can be drawn from the data by summarizing the main findings of the studies conducted in this domain. Here attention is divided between discussion of the evidence regarding the role of GSH in the context of mental illness and the utility of MRS in the measurement of this metabolite in vivo.

#### 3.1.2 Findings of in vivo GSH and mental illness

The majority of the studies (37) were aimed at determining if GSH concentration significantly differed between healthy cohorts and those with diagnosed mental disorders, or if there were specific alterations by diagnostic sub‐group. A second group of studies (7) were targeted at novel approaches to spectral measurement and quantification yet used in vivo measurements of GSH concentration both as a test‐bed for novel techniques as well as a means of confirmation of earlier findings. Eighteen of the studies concentrated (at least in part) on the anterior cingulate cortex (ACC), while the remainder contained a diverse range of regions of interest from the medial (m), dorsal (d) and posterior (p) PFC (11 studies), hippocampus (Hip: three), occipital cortex (OCC: four), temporal lobes (Temp: two) and one study each from the basal ganglia (BG), the thalamus (Th), the midline parietal, the precuneus (Pre), the left insular LI) and the visual cortex (VI) with additional studies that performed spectroscopy over an array of voxels, or chemical shift imaging (CSI). With the exception of CSI, regions were studied using a single large voxel (of varying size, but generally around 2 × 2 × 2 cm^3^). Voxels were placed and oriented by a researcher, guided by a scout scan over the region of interest, and multiple regions were studied with separate voxels acquired sequentially.

Any patterns of GSH perturbation are difficult to identify, since there are only a few relevant studies that met our criteria for this review.

Overall, evidence of altered GSH concentration was inconsistent. While there were some indications of a decrease in GSH for major depression, treatments did not alter GSH levels—even when those treatments were associated with decreased symptoms. However, these changes are consistent with the changes seen in blood GSH levels (Maes, Galecki, Chang, & Berk, 2011), providing some support for a true alteration of GSH systemically. While generally there was some evidence that GSH levels are perturbed in first‐episode psychosis, there was no consistency in the direction of change, and no changes were observed in either chronic schizophrenia patients or individuals at clinical high risk for psychosis.

None of the studies of BD included in this review show a change in brain GSH compared with controls. This may reflect a medication effect, since lithium (a commonly prescribed mood‐stabilizer) has antioxidant properties and most notably can increase GSH in rat cerebral cells (Cui, Shao, Young, & Wang, 2007). Another interesting point to consider is the difference between brain GSH and blood GSH in BD. Studies present conflicting blood and brain GSH data, suggesting that blood GSH levels are abnormal or perturbed (Gu, Chauhan, & Chauhan, 2015). This may represent a compensatory response of GSH production in the brain to balance the depleted peripheral GSH pool, or may suggest that the two systems are locally regulated via the GSH‐GSSG cycle.

There were some correlational findings between GSH concentration and clinical variables. For example, in early psychosis, GSH concentration in the posterior medial frontal cortex was negatively correlated with negative symptoms of schizophrenia (Berger et al., 2008; Demro et al., 2017). Negative symptoms are typically more difficult to treat with traditional anti‐psychotic medication, and this relationship suggests that an intervention to increase GSH may be a promising alternative treatment. Similarly, in MD, there was a negative relationship between GSH and anhedonia. While there were relationships in bipolar disorder between GSH and risky drinking (based on the alcohol use disorders identification test; Chitty, Kaur, Lagopoulos, Hickie, & Hermens, 2014; Chitty, Lagopoulos, , Hickie, & Hermens, 2014; Saunders et al., 1993) or smoking. Finally, as a function of age, a positive relationship was observed between GSH and AD, MCI and cognitive decline, yet these observations may simply be due to other age‐related effects.

#### 3.1.3 Heterogeneity of studies and the strength of a brain GSH measurement

The complex nature of psychiatric illness, as well as the difficulty in GSH measurement, leaves a heterogeneous evidence base that makes comparison challenging. Contradictions between studies assessing brain GSH make it difficult to compare brain and blood GSH, where studies assessing blood GSH present consistent findings in perturbation in GSH metabolism for first‐episode psychosis (Fraguas, Díaz‐Caneja, Rodríguez‐Quiroga, & Arango, 2017), schizophrenia (Ng, Berk, Dean, & Bush, 2008), bipolar disorder and autism (Gu et al., 2015).

##### Medication exposure

Medication status is important to consider as a confounder when assessing oxidative stress and GSH antioxidant action within youth mental health. In bipolar disorder, lithium and valproate (commonly prescribed mood stabilizing drugs) results in a dose‐dependent GSH increase in rat cortical cells. After 1‐week chronic treatment, the cells demonstrated reduced oxidative stress, including increased GSH and GCL expression (upstream GSH synthesis; Cui et al., 2007). This presents a new issue, here GSH is elevated as a result of drug administration, rather than as a result of illness. It is possible the cessation of the medication would result in a return to the baseline levels of GSH. Another study looking at first‐episode mania in bipolar disorder (Machado‐Vieira et al., 2007) assessed the effects of lithium treatment vs no‐treatment. Antioxidants SOD and CAT were elevated in unmedicated patients, indicative of generation of reactive species intrinsic to the illness. Lithium reduced markers of oxidative stress (TBARS‐lipid damage), as well as the SOD/CAT ratio. There is a small body of evidence associating anti‐psychotic and mood‐stabilizing medication with protective effects such as increased antioxidant enzyme activity or expression, and increased GSH (Cui et al., 2007; Wang, Xu, Dyck, & Li, 2005). This may have a profound effect on studies in early psychosis, since not all studies control for drug‐naïve vs medicated patients. The use of a first generation dopamine antagonist like haloperidol may contribute to increased oxidative injury (Lohr, Kuczenski, Bracha, Moir, & Jeste, 1990). In patients with chronic schizophrenia treated with haloperidol, increased lipid peroxidation markers were observed (TBARS), as well as the antioxidant enzyme SOD, presumably in response to the peroxidative injury to membrane phospholipids (Gama, Salvador, Andreazza, Kapczinski, & Silva Belmonte‐de‐Abreu, 2006). There are some second‐generation dopamine antagonists that demonstrate protective properties. Olanzapine, clozapine, quetiapine and risperidone all play a role in upregulating SOD1 gene expression (Bai et al., 2002), indicative of an antioxidant response against radical species. The most common treatment for depression, selective‐serotonin reuptake inhibitor prescription, has resulted in significant reductions in lipid peroxidation as a marker of oxidative stress (Khanzode, Dakhale, Khanzode, Saoji, & Palasodkar, 2003). This study found that treatment with fluoxetine and citalopram decreased serum SOD (antioxidant) and MDA (lipid peroxidation marker).

##### Voxel placement

Because there are few studies analysing GSH levels in mental illness using MRS, there appears to be no “gold standard” voxel location. There is rationale for the use of the medial frontal cortex due to association of this region with schizophrenia (Pomarol‐Clotet et al., 2010). It has been argued that the medial temporal lobe is a more appropriate location for spectroscopy since there are reported links with schizophrenia, as well as the regions vulnerability to insult, particularly in the context of oxidative stress (Wood, Yücel, et al., 2009). The concentration of GSH in different regions of the brain varies significantly. The studies that have considered brain GSH have focused on the cortex, which represents the greatest concentration of brain GSH, with the cerebellum, hippocampus and striatum following in descending order of GSH concentration (Kang et al., 1999). The location of GSH in the brain is highly tissue specific (Rae & Williams, 2017), with a report of 30% higher GSH in cortical white matter (WM) compared with grey matter dominated PFC (An et al., 2015). Grey matter (GM) demonstrates increased metabolite concentrations (Srinivasan, Ratiney, Hammond‐Rosenbluth, Pelletier, & Nelson, 2010), and this tissue also consumes oxygen in a 4:1 ratio to WM, despite only comprising 40% of the total brain volume (Mintun et al., 2001), rendering the GM more susceptible to oxidative insult. When comparing the frontal cortex of young healthy participants, there was a significantly greater concentration of GSH in females compared with males (Mandal et al., 2012), which could provide a confounding factor when comparing both genders in youth mental health. These results indicate a significant gender bias towards GSH concentration in the brain, which may be important considering the greater incidence of schizophrenia and first‐episode psychosis (Ochoa, Usall, Cobo, Labad, & Kulkarni, 2012) in males.

##### Tobacco and cannabis

The prevalence of smoking in people with mental illness far outweighs that in the general population. It is estimated in the United Kingdom that 16% of the general population smoke, whereas in psychosis incidence is 56%, major depression 40%, anxiety 37% and OCD 40% (Szatkowski & McNeill, 2013). Exogenous administration of nicotine to isolated cell lines in vivo reduces antioxidant constituents (Yildiz, Liu, Ercal, & Armstrong, 1999), including GSH. Within a chronic schizophrenia population, tobacco smoke induces the oxidation of lipids and proteins (Yao, Leonard, & Reddy, 2006). In bipolar disorder, GSH concentration is reduced in the ACC of smokers, but not the non‐smoking patients (Chitty, Lagopoulos, et al., 2014), and in a longitudinal study assessing tobacco consumption, a reduction in smoking was a significant predictor of increased GSH (Chitty, Lagopoulos, Hickie, & Hermens, 2015a, 2015b).

In marijuana research, it has been reported that brief exposure of an endothelial cell line to marijuana (3.95%) stimulated increased oxidative stress by 80%, as well as an 81% reduction in GSH concentration (Sarafian, Magallanes, Shau, Tashkin, & Roth, 1999). In the same study, exposure to smoke containing no THC (psychoactive component of cannabis) resulted in no change in oxidative species, but a decline in GSH of 70%. This is interesting since, as with tobacco, marijuana use in mental illness is far greater than in the general population (6.6% (UNDOC, World Drug Report, 2011) compared with 23% in psychosis (Green, Young, & Kavanagh, 2005), 9.5% in major depression (Chen, Wagner, & Anthony, 2002), 19% in bipolar disorder (Marken et al., 1992) and 17% in anxiety (Degenhardt, Hall, & Lynskey, 2001)).

### 3.2 Strength of a brain GSH measure over periphery

A number of studies have tried to draw comparisons between GSH in the brain and peripheral measures such as blood components or cerebrospinal fluid (CSF). Due to the variable nature of study design in the tissue assessed and the brain region of interest, the concentration of GSH across differing compartments of the human body is not well characterized (Richie, Skowronski, Abraham, & Leutzinger, 1996; Samuelsson, Vainikka, & Ollinger, 2011; Sanaei Nezhad, Anton, Parkes, Deakin, & Williams, 2017)).

Many studies, when investigating GSH, do not differentiate between reduced, oxidized and total GSH, rendering the measure comparable only within its own intervention, and difficult to use in comparison to other studies. This makes the use of MRS as a tool for GSH quantification useful since the measure is direct, and informative of brain GSH status.

Blood measures of GSH are the most commonly reported methods, since assays are cheaper and more accessible than MRS. Plasma measures of GSH are commonly reported, but report a wide range of concentrations of GSH (0.5‐759 μM) (Nuttall, Martin, Sinclair, & Kendall, 1998; Raffa et al., 2009). Plasma acts as a medium for metabolic waste products in the body, where cells excrete partial protein and lipid components for clearance. In addition, the role of GSH is to provide redox balance within a cellular system. Therefore, it is unlikely that plasma GSH offers a reliable and accurate representation of peripheral GSH, and even more unlikely that it would be a representation of brain GSH.

### 3.3 MRS methods

MRS provides a means to explore novel chemical environments and analyse known molecular systems. Through the analysis of the free induction decay (FID) spectra of a target nucleus, the local chemical environment may be inferred. While exotic nuclei, such as ^31^P and ^13^C, are possible for targeting in clinical environments, ^1^H is the most widely available (Blüml, 2013). However, accurate quantification of some metabolites is hampered by both the low spatial resolution, due to the low chemical concentrations and the decreased spectral resolution of clinical scanners with lower field strengths. Generally, in vivo MRS is conducted over a single voxel placed within a target brain region (identified using a preliminary scout scan); while imaging protocols that perform spectroscopy over an array of large voxels are possible they are less common.

### 3.4 GSH measurement in vivo

In vivo quantification of GSH in humans is particularly challenging due to the low concentration (1.5‐3 mmol/L), and the fact that all resonances overlap with stronger signals from alternate metabolites (De Graaf, 2013). However, it is possible to quantitatively isolate the spectral contribution from GSH through both refinement and calibration of standard approaches to MRS measurement as well as the development of novel sequences. Additionally, in order to accurately quantify the contribution of GSH to the measured spectra, a range of post‐processing techniques, from spectral decomposition to partial volume correction, are required. While similarities and trends do exist, studies conducted within the past 10 years vary in voxel placement, measurement sequence, acceptance bounds on signal quality and post‐processing methodologies. Here the predominant approaches are described.

### 3.5 Acquisition modalities

In order to accurately estimate the concentration of GSH within a volume of interest, a range of different tradeoffs must be considered. The method of spatial localisation utilized is, perhaps, the first consideration, while voxel size and placement, which is affected by field and tissue homogeneity, are of related concern as are the methods used to isolate GSH from other contributors.

#### 3.5.1 Spectroscopy

The FID spectrum exhibits a range of peaks unique to the local electronic environment of contributing chemical systems. In order to quantify low concentration metabolites, the extracted spectra must be of high quality, predominantly assessed through the signal‐to‐noise ratio (SNR) and the spectral linewidth (usually expressed in terms of the full width at half maximum—FWHM). While quantification accuracy is usually dependant on the spectral SNR, small linewidths allow for good separation of distinct peaks. Averaging multiple signals can increase the SNR, yet patient motion means that shorter scan times are preferred (both in terms of field homogeneity and partial volume correction). Approaches to decomposition using basis vectors determined by contributing metabolites can decrease the importance of linewidth (removing any requirement of spectral separation), yet the accuracy to which each contribution can be estimated still relies on this parameter.

A high‐quality shim can be used to provide field (B0) homogeneity over some region of interest, reducing the expected spectral linewidth sufficiently for GSH analysis. However, specific sequences are required in order to localize the induced FID to this region. A number of different approaches exist, each with competing properties in terms of measured spectral quality.

#### 3.5.2 Spatial selectivity

**Figure 2:**
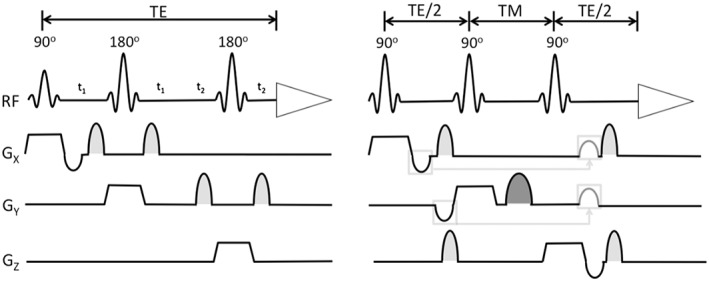
Point‐resolved spectroscopy (right) and stimulated echo acquisition mode (left) sequences, taken from “In Vivo NMR Spectroscopy,” (De Graaf, 2013)

While a range of spatial localisation methods exist, the studies considered here used point ‐resolved spectroscopy (PRESS) or stimulated echo acquisition mode (STEAM), and variants or extensions to these sequences, almost exclusively.^2^ PRESS and STEAM both use three slice selective pulses (Figure 2):

1. PRESS: 1 × 90^o^ + 2 × 180^o^
2. STEAM: 3 × 90^o^

STEAM decreases the specific absorption and because there is no T2 decay in the TM period is preferred for short T2 metabolites, but this approach does sacrifice the SNR of each acquired spectra.

#### 3.5.3 Water suppression and spectral editing

Because of the local environment, water suppression is required in order to resolve the much smaller spectral peaks induced by the target metabolites. Chemical shift selective and variable pulse powers and optimized relaxation delays (VAPOUR) are commonly used for water suppression, while it is also possible to include water into the macromolecular baseline utilized during molecular decomposition.

With an accurate high‐order shim, it is possible to resolve GSH peaks directly. However, should the spectral linewidth be too large (or simply to enhance quantitative accuracy) spectral editing may also be used to extract specific metabolic signals. The MEscher‐GArwood (MEGA) technique uses editing pulses aimed at spins which are J‐coupled with the spin of interest, for instance, an editing pulse is applied at 4.56 ppm which targets a α‐CH resonance J‐coupled to the desired GSH spins at 2.95 ppm (Saleh et al., 2016). Sequences with and without editing are subtracted to produce a final spectrum that contains greatly decreases cross‐talk between the spins of interest and other peaks within the spectra.

#### 3.5.4 Modifications and extensions

The predominant approach to spectral measurement in the studies considered here used highly optimized PRESS and STEAM sequences directly with, in some cases, editing sequences such as MEGA to home in on specific metabolites. However, a range of those studies considered also investigated novel spectroscopy acquisition protocols. Such approaches used relationships involving GSH, tested in vivo, to not only validate and test the novel protocol, but also to validate and explore the metabolic relationships.

- **Hadamard encoding and reconstruction of mega‐edited spectroscopy:** The use of a Hadamard encoding in editing allows the simultaneous acquisition of two different voxels and two different metabolites simultaneously, quartering acquisition time (or doubling SNR).
- **Phase rotation**: Phase cycling is introduced into the radio frequency signal to remove undesired spin echo signals, and has been used with STEAM to isolate the stimulated echo.
- **2D J‐differences editing**: Two dimensionally resolved MRS provides spectra over a range of editing frequencies and as such can be used to perform accurate MRS with no expectation as to the target metabolite.
- **MRS imaging:** While direct repetition of PRESS or STEAM for multiple voxels is possible, imaging (or spatial encoding of the spectroscopic signal) is generally conducted using phase encoded gradient fields.
- **Proton echo planar spectroscopic imaging (PEPSI):** PEPSI uses an approach to echo planar imaging, yet one dimension of echos are encoded with chemical shift information, rather than spatial information as in standard EPI. In studies considered that employ PEPSI large outer volume suppression slabs and advanced approaches to shimming (FAST(EST)MAP) were required.

### 3.6 Data acquisition post‐processing

Once the spectra have been acquired, extraction of the GSH signal is required. Signal extraction can take a number of directions and may be dependent on the approach taken to measurement. Should sufficient and accurate editing have been conducted, the GSH peak may then be measured directly (often using simple summation over the GSH peak). However, in the case that there is residual overlap between GSH and spectra from other metabolites present in the sample more sophisticated processing is required.

#### 3.6.1 Spectral deconvolution

A standard approach, in the case of overlapping peaks, is linear decomposition of the acquired spectra into metabolic basis functions. Standard software packages (such as LCModel) are available, or in‐house software may also be used for this approach. In all cases, a database of metabolic basis functions must be available that are either carefully measured (with the same system and sequence used for measurement), simulated using advanced modelling tools such as GAMMA, VERSI, FID‐A (or in‐house developed tools) or even a combination of the two where measurements of the macromolecular spectra may be measured and used in concert with simulated spectra of specific metabolites.

Once the basis metabolites are identified, they are used to estimate a linear decomposition of the measured spectra (based on some cost function such as least‐squares). The decomposition provides an estimate of the contribution from each metabolite, while the residual allows for the presence of missing features to be assessed (ie, it should represent noise). Generally, such fits also provide a measure of uncertainty, as a percentage of the Cramer Rao lower bound (CRLB) in the case of LCModel, which can be used to accept or reject the measurement. Where acceptance levels were quoted as CRLB, most commonly >20% uncertainty measurements were rejected, but in some cases rejection was reserved only for >50% uncertainty. While it is not clear as to the impact on final values of the estimated value or its uncertainty, nor rejection levels utilized, using the %CRLB as a rejection threshold has been shown to be unreliable, particularly for low concentration metabolites (Kreis, 2016). Kreis (2016) argued that despite the unreliability of these widely used threshold levels of 20% to 50%, CRLB is a valuable tool to give an idea of minimal uncertainties in MRS, when the obtained error is understood to be an estimate of the lower bound of the fitting error.

Finally, once the metabolite concentration has been identified, the voxel heterogeneity was accounted for (in a similar way for most studies). The MRS voxel (or voxels) used in measurement is segmented based on the original magnetic resonance imaging image used for positioning. The contributing volume fractions from GM, WM and CSF are calculated (often using the FAST4 algorithm from FSL) and used to correct the measured concentration (or understood as covariates with this concentration).

#### 3.6.2 Measurement normalization and post‐processing

Some note should be made regarding the comparison of spectroscopy techniques across the studies considered here. However, this is made difficult not only by the broad range of sequences used but also the range of parameters explored within each sequence. In each study different spectral bandwidths, sampling rates, and number of averages were considered. Each of these parameters will affect the FWHM and SNR of the recovered spectra, and hence the reliability of the spectral decomposition (as will the methods used to generate the basis metabolites). Even the units used in measurement of the metabolic concentration differed. While in many studies the absolute GSH concentration was used as the final measurement, in other studies GSH as a fraction of total Creatine (tCr) or Water peaks were used. Finally, comparison across studies that use different scanners is a potential confound, while the pooling of MRS data is becoming more important in terms of providing a larger sample size to increase confidence in effect size, or to facilitate collaborative work, the risk of systematic error is increased, since there is always variability in participant positioning, partial volume effects and image intensity inhomogeneity (Stonnington et al., 2008).

Such diversity may not impact the overall neuroscientific findings, yet each choice will impact the accuracy of each finding, hindering comparison. No study regarding the impact on quantification of the relevant tradeoffs has been conducted, so that generally in‐house optimisations and conveniences have been used. While useful for immediate application, this approach may hinder a standardized approach for quantitative exploration.

## 4 SUMMARY

There is evidence of perturbations in the oxidative stress/antioxidant response system across youth metal health, with emerging evidence implicating GSH in the eitiology of mental illness. However, the field is hampered by highly variable methodology, from basic sequence and voxel selection to analysis approach and choice of measurement units. This is before problems surrounding diagnostic heterogeneity are considered and the simple issue of statistical power. If this area of study is to progress, studies with larger participant numbers are required, most probably involving multiple collaborating sites where very close attention is paid to minimizing between‐site variability in both clinical and spectroscopic data collection.

## ENDNOTES

1 Adapted from http://www.djmannion.net/psych_programming/data/power/power.html

2 Exceptions include spin‐echo full‐intensity acquired localized spectroscopy (SPECIAL) and imaging studies.
